# Curcumin Attenuates Ferroptosis-Induced Myocardial Injury in Diabetic Cardiomyopathy through the Nrf2 Pathway

**DOI:** 10.1155/2022/3159717

**Published:** 2022-07-15

**Authors:** Zhang Wei, Qian Shaohuan, Kang Pinfang, Shi Chao

**Affiliations:** ^1^Department of Cardiovascular Medicine of the First Affiliated Hospital of Bengbu Medical College, Bengbu City, Anhui, China 233000; ^2^Department of Cardiac Surgery of the First Affiliated Hospital of Bengbu Medical College, Bengbu City, Anhui, China 233000

## Abstract

Diabetes causes lipid peroxide to accumulate within cardiomyocytes. Furthermore, lipid peroxide buildup is a risk factor for ferroptosis. This study is aimed at examining whether curcumin can ameliorate ferroptosis in the treatment of diabetic cardiomyopathy. Hematoxylin and eosin and Masson sections were used to examine the morphology, arrangement, and degree of fibrosis of the myocardium of diabetic rabbit models. The expression levels of nuclear Nrf2, Gpx4, Cox1, and Acsl4 in diabetic animal and cell models were quantitatively analyzed using immunofluorescence and western blotting. Nrf2-overexpression lentivirus vectors were transfected into cardiomyocytes, and the protective effects of curcumin and Nrf2 on cardiomyocytes under high glucose stimulation were assessed using terminal deoxynucleotidyl transferase dUTP nick-end labelling and reactive oxygen species probes. Diabetes was found to disorder myocardial cell arrangement and significantly increase the degree of myocardial fibrosis and collagen expression in myocardial cells. Curcumin treatment can increase nuclear transfer of Nrf2 and the expression of Gpx4 and HO-1, reduce glucose induced myocardial cell damage, and reverse myocardial cell damage caused by the ferroptosis inducer erastin. This study confirmed that curcumin can promote the nuclear translocation of Nrf2, increase the expression of oxidative scavenging factors, such as HO-1, reduce excessive Gpx4 loss, and inhibit glucose-induced ferroptosis in cardiomyocytes. This highlights a potentially new therapeutic route for investigation for the treatment diabetic cardiomyopathy.

## 1. Introduction

Diabetes is a worldwide chronic metabolic condition. Serious complications affecting the nerves and blood vessels can occur as the course of diabetes progresses. Specifically, cardiovascular disease is one of the most common clinical complications of diabetes [1]. Elevated blood sugar levels can lead to lipid metabolism problems among vascular endothelial cells and cardiomyocytes and the accumulation of local inflammatory factors. This leads to coronary stenosis, myocardial fibrosis, and myocardial systolic dysfunction. Subsequently, severe heart failure can develop, lowering the long-term quality of life of these patients [2].

Ferroptosis is a necrotic inflammatory reaction caused by the accumulation of intracellular peroxides brought on by a variety of inflammatory factors and lipid metabolism disorders [3]. According to previous research, pancreatic beta cells express low levels of antioxidant enzymes making them prone to excess reactive oxygen species (ROS) accumulation which can trigger a variety of inflammatory responses. When in vitro human pancreatic beta cells are treated with the ferroptosis inducer erastin, glucose-stimulated insulin secretion capacity is significantly reduced [4]. Furthermore, during diabetic cardiomyopathy, cardiomyocyte cells obtain almost all of their energy from fatty acid oxidation resulting in excessive fatty acid oxidation, accumulation of peroxides and inflammatory factors, and irreversible cardiomyocyte damage [5, 6].

Curcumin is a polyphenolic compound extracted from the rhizomes of the turmeric plant and has a wide range of biological activities, including free radical scavenging and antioxidant, anti-inflammatory, and anticancer properties [7, 8]. According to Wang et al., curcumin can reduce aflatoxin B1-induced liver damage by regulating pyroptosis and the nuclear factor erythroid-2-related factor 2 (Nrf2) signaling pathway [9]. Nrf2 is one of the most important regulators of antioxidative stress in the body. By regulating the expression of glutathione synthesis and nicotinamide adenine dinucleotide phosphate generation, the Nrf2/ARE signaling pathway can neutralize the accumulated superoxide and inhibit oxidative stress [10, 11]. Therefore, this study is aimed at investigating whether curcumin can regulate Nrf2 expression and alleviate ferroptosis-induced myocardial injury in diabetic animal and cellular models.

## 2. Materials and Methods

### 2.1. Construction of Diabetic Rabbit Model

Two-month-old male New Zealand rabbits purchased from the Medical Experimental Animal Center of Bengbu Medical College were used as experimental subjects. Streptozotocin was dissolved in sterile saline and intraperitoneally injected into the rabbits at a dose of 80mg/kg. The rabbits were allowed to eat freely after receiving the injection. The fasting blood glucose levels of the rabbits were monitored regularly. The diabetic rabbit model was considered successfully established when the fasting blood glucose level was measured as 11 mmol/L twice or 14 mmol/L once. Following successful modelling, grouping was performed as follows: blank control group (Con-Group), diabetic rabbit group (DM-Group), diabetic rabbit + every other day curcumin administration group (Qod-Group), and diabetic rabbit + daily administration group (Qd-Group). Six rabbits were randomly assigned to each experimental group and raised in the same environment for 3 months. A curcumin solution was prepared using 1% sodium carboxymethyl cellulose for oral administration through drinking water at a concentration of 300 mg·kg^−1^·d^−1^. The animals were kept in an animal room at the Bengbu Medical College at 25°C with 12 h light-dark cycles. The environment was regularly cleaned by professionals, and each group was raised in separate cages. This study was approved by the Medical Ethics Committee of the First Affiliated Hospital of Bengbu Medical College (2022:031).

### 2.2. Rabbit Myocardial Slices

All animals were anaesthetized using ether and fixed on the operating table. They were euthanized with intravenous propofol and potassium chloride. Then, the heart was removed and the blood thoroughly washed away with PBS. Myocardial tissue of the apex of ~1 cm was cut, fixed with 4% paraformaldehyde, and embedded in paraffin. The embedded myocardial tissue was sectioned using a microtome, and hematoxylin and eosin and Masson staining was performed to analyze myocardial fibrosis and morphology. The paraffin sections were fluorescently stained with Gpx4 and collagen II antibodies to determine protein expression levels.

### 2.3. Grouping of Cardiomyocytes

Rat H9C2 cardiomyocytes were used as experimental cell models (long-term culture cryopreserved in the laboratory). Cells were either treated with a normal glucose concentration medium of 5.5 mmol/L (Nor-Group) or a high-glucose concentration medium of 30 mmol/L (H-Group).

### 2.4. CCK-8 Measured Cell Viability

The H9C2 cardiomyocytes were cultured in 96-well plates. When they reached ~70% confluency, different concentrations of curcumin were added and the cardiomyocytes were cocultured for 24 h. CCK-8 reagent was added, and relative cell activity was measured using a microplate reader to determine the optimal curcumin concentration. Three duplicate wells were set up for each group.

### 2.5. Nrf2 Virus Construction and Transfection

The Shanghai Jikai Company in China provided Nrf2 viral vectors. When the cardiomyocytes had reached ~70% confluence, an appropriate amount of viral vector was mixed into 3 mL of fresh medium containing 6 *μ*g/mL of polybrene. The cardiomyocytes were cultured in the medium, and transfection efficiency was assessed using a fluorescence microscope. For multiple transfections, the best virus transfection multiplicity of infection value was used. Transfection efficiency was verified using western blotting (WB).

### 2.6. Extraction of Nuclear Proteins

After cell digestion and centrifugation, an appropriate amount of cytoplasmic protein extraction reagent was added, thoroughly mixed, and placed in an ice bath for 10 min before centrifuging at 12000 *g* for 15 min to remove the supernatant. Then, the required amount of nuclear protein extraction reagent was added, thoroughly mixed, placed in an ice bath for 10 min, and centrifuged at 12000 *g* for 15 min. The supernatant was collected and used to measure nuclear protein.

### 2.7. Main Experimental Reagents

Curcumin (Sigma, *America*); Nrf2 virus vectors (Gikai, Shanghai, China); Heme Oxygenase 1 Rabbit Polyclonal Antibody, Cox1 Rabbit Polyclonal Antibody, Nrf2 Rabbit Polyclonal Antibody, Gpx4 Rabbit Polyclonal Antibody, One-Step TUNEL Apoptosis Assay Kit, Reactive Oxygen Species Assay Kit (Beyotime Biotechnology, Shanghai, China); Acsl4 (Abcam, United Kingdom); Dylight 594-Goat Anti-Rabbit IgG (Abbkine Scientific, Wuhan, China); DMEM high-sugar medium (Hyclone, *American*); and Nuclear Protein Extraction Kit (Solarbio Life Science, Beijing, China) were used.

### 2.8. Statistical Analysis

SPSS 25.0 software was used to process all statistical data. All experiments were conducted at least three times, and data are expressed as mean ± SD. *t*-tests were used for data analysis between two groups. Images were analyzed using ImageJ software. Statistical significance was set at a *P* value of <0.05.

## 3. Results

### 3.1. Myocardial Tissue Sections and Western Blotting

**Figure 1:**
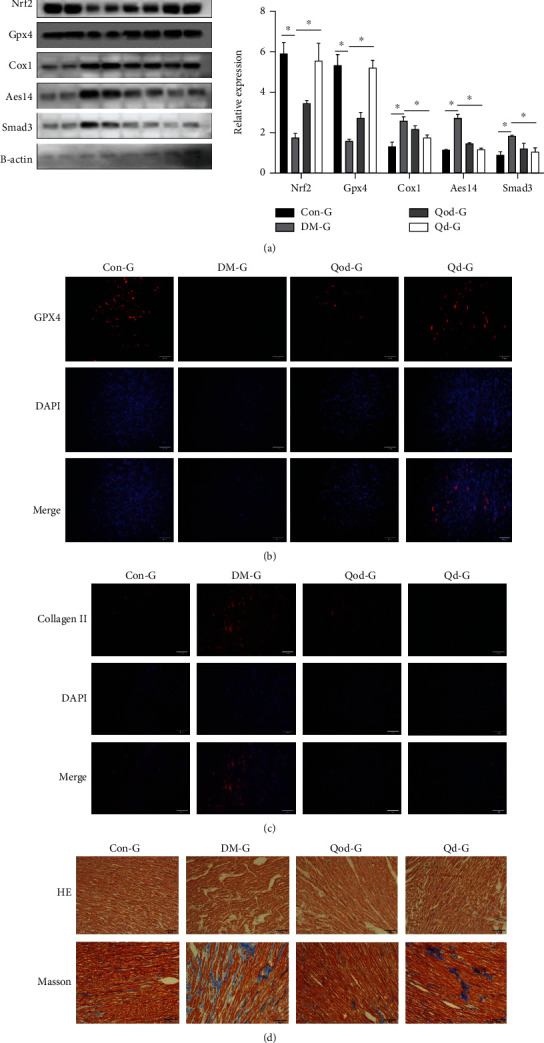
Curcumin inhibits ferroptosis and improves myocardial fibrosis. (a) The expression levels of ferroptosis-related proteins in each group were detected by western blotting. The results showed that curcumin significantly inhibited the expression of ferroptosis-related proteins (^∗^*P* < 0.05). Data are expressed as mean ± SD. (b) Immunofluorescence staining of rabbit myocardium for Gpx4 protein expression (×200). (c) Collagen immunofluorescence staining in rabbit myocardial tissue (×200). (d) Hematoxylin and eosin and Masson staining of rabbit myocardial tissue demonstrating how curcumin significantly inhibits glucose-induced myocardial fibrosis (×200).

According to WB results, diabetes increased the expression of cyclooxygenase 1 (Cox1) and fatty acid coenzyme A ligase (Acsl4) proteins in the ferroptosis pathway in myocardial tissue (Figure 1(a)). Immunofluorescence results revealed that Gpx4 protein levels were significantly reduced in rabbit model diabetic myocardial tissue (Figure 1(b)). In contrast, curcumin reduced the expression of ferroptosis-related proteins in myocardial tissue (Figures 1(a) and 1(b)). Hematoxylin and eosin staining revealed that curcumin greatly improved disordered myocardial cell arrangement brought on by diabetes, alleviated myocardial cell edema, and decreased lipid accumulation between tissues. Masson's staining and tissue collagen fluorescence data confirmed that diabetes increased myocardial fibrosis (Figures 1(c) and 1(d)). However, curcumin decreased the expression of collagen in myocardial tissue, reducing the degree of myocardial fibrosis (Figures 1(c) and 1(d)).

### 3.2. Curcumin Reduces Cell Damage Caused by High Glucose Levels

**Figure 2:**
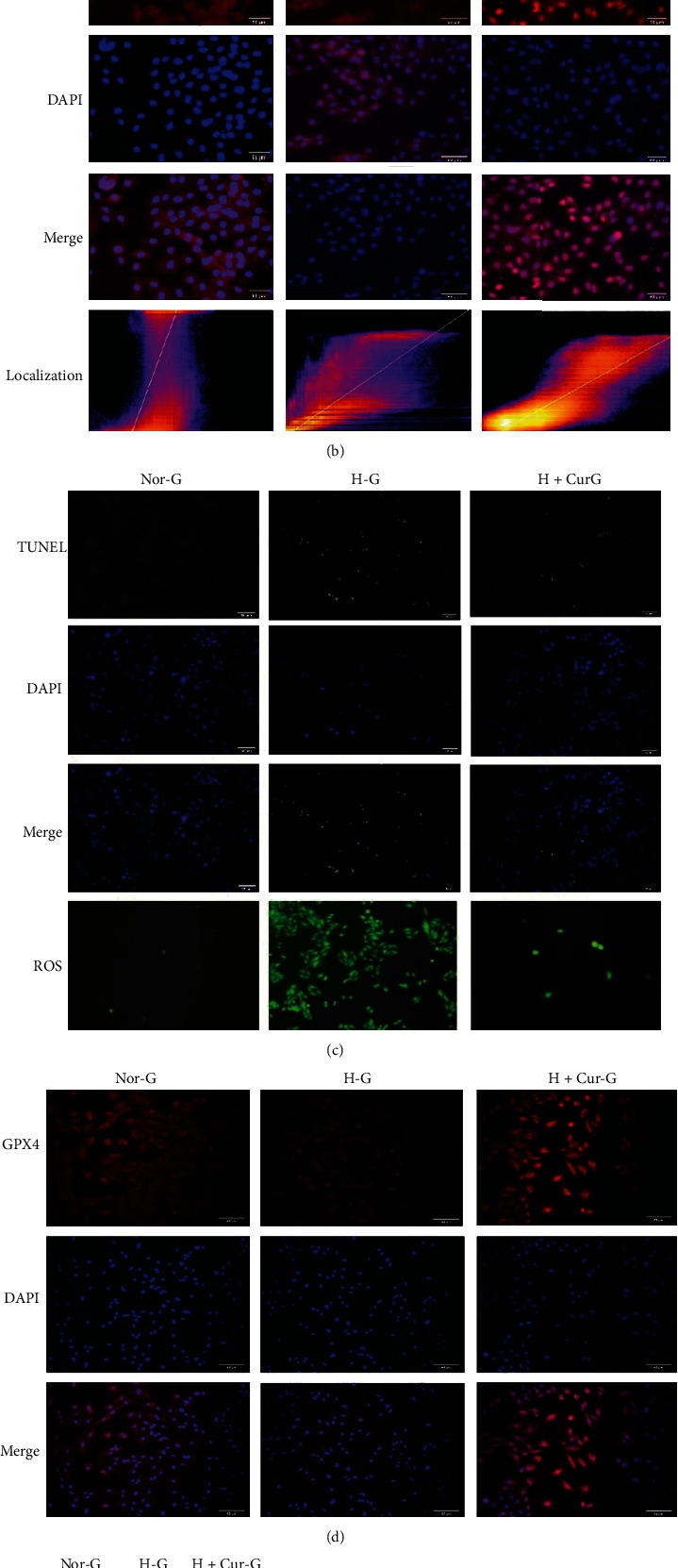
Curcumin improves myocardial cell damage. Nor-G: 5.5 mmol/L glucose concentration; H-G: 30 mmol/L glucose concentration; H-Cur-G: 30 mmol/L glucose concentration + curcumin. (a) CCK-8 assay detected myocardial cell activity at different curcumin concentrations. (b) Expression level and localization of Nrf2 assessed by immunofluorescence (×200). Fluorescence colocalization analysis showed that curcumin induced Nrf2 nucleus localization with a Pearson correlation coefficient of 0.59, indicating a strong correlation. (c) Apoptosis and intracellular ROS levels were measured using TUNEL and ROS fluorescent probes (×200). (d) Gpx4 cell expression was detected using immunofluorescence (×200). (e) The expression levels of Nrf2 protein in the nucleus and ferroptosis pathway-related proteins were detected by western blot (^∗^*P* < 0.05).

In the CCK-8 assay, the optimal curcumin concentration was found to be 10 *μ*mol/L (Figure 2(a)). The cells in each group were cocultured for 24 h. Fluorescence colocalization was used to examine Nrf2 expression in the nucleus which revealed that curcumin promoted Nrf2 nuclear translocation. The Pearson correlation coefficient was 0.59 indicating a strong association between the two fluorescence groups (Figure 2(b)). WB reinforced that curcumin promoted nucleus translocation of Nrf2 (Figure 2(e)). Intranuclear transfer of Nrf2 increased the expression of antioxidant factor Heme Oxygenase-1 (HO-1), reducing cardiomyocyte accumulation of intracellular oxides, alleviating Gpx4 depletion, and inhibiting the progression of glucose-induced ferroptosis (Figures 2(c)–2(e)).

### 3.3. Nrf2 Overexpression Reduced Glucose-Induced Cell Injury

**Figure 3:**
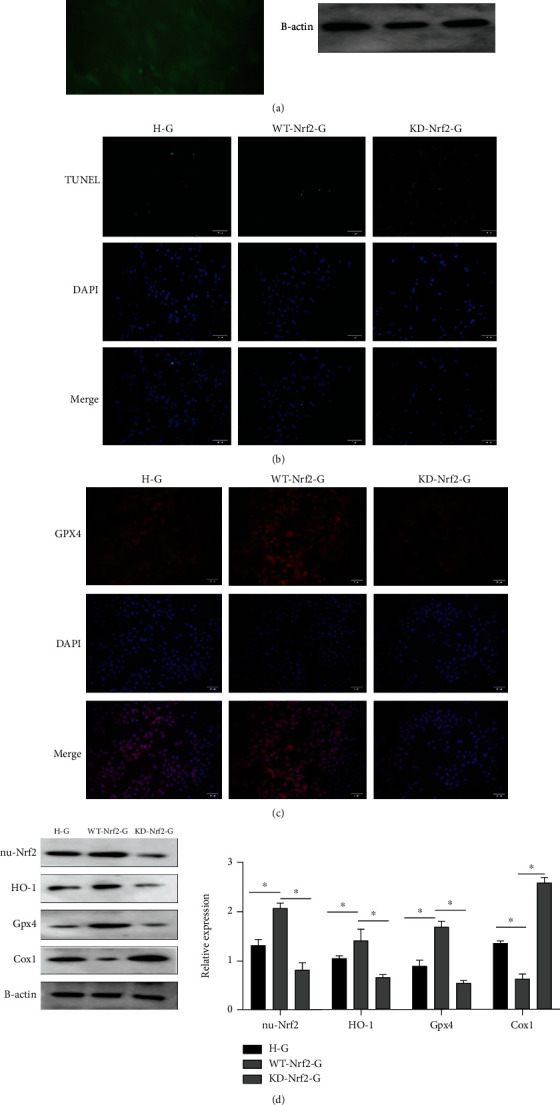
High glucose-induced cardiomyocyte damage can be alleviated by overexpression of Nrf2. H-G: 30 mmol/L glucose concentration; WT-Nrf2-G: Nrf2 overexpressing+30 mmol/L glucose concentration; KD-Nrf2-G: knockdown of Nrf2 expression+30 mmol/L glucose concentration. (a) Virus transfection efficiency was determined using fluorescence, and the optimal multiplicity of infection value was 20 (×200). Western blot confirmed that Nrf2 was overexpressed and successfully knocked out among the experimental groups (*P* < 0.05). (b) The apoptosis rate of cardiomyocytes in each group was detected using terminal deoxynucleotidyl transferase dUTP nick-end labelling (TUNEL) fluorescent probe. Overexpression of Nrf2 significantly alleviated myocardial death induced by high glucose concentrations (×200). (c) Immunofluorescence was used to detect the level of Gpx4 expression in each group. Overexpression of Nrf2 significantly reduced GPX4 depletion (×200). (d). Western blot assays for expression of related factors among the different groups. Transfection with Nrf2 viral vectors increased the expression of Nrf2 in the nucleus (^∗^*P* < 0.05).

Cardiomyocyte Nrf2 expression was assessed using WB after lentivirus transfection (Figure 3(a)). Nrf2 nuclear overexpression dramatically increased the expression of the downstream antioxidant factor HO-1 (Figures 3(c) and 3(d)). HO-1 and Gpx4 protein expression was dramatically reduced in the knockdown group; however, Cox1 protein expression in ferroptosis was significantly elevated which led to decreased cell activity in this group (Figures 3(b)–3(d)). Therefore, overexpression of Nrf2 has the effect of inhibiting myocardial injury caused by high glucose.

### 3.4. Curcumin and Nrf2 Protect against Ferroptosis-Inducing Agents

**Figure 4:**
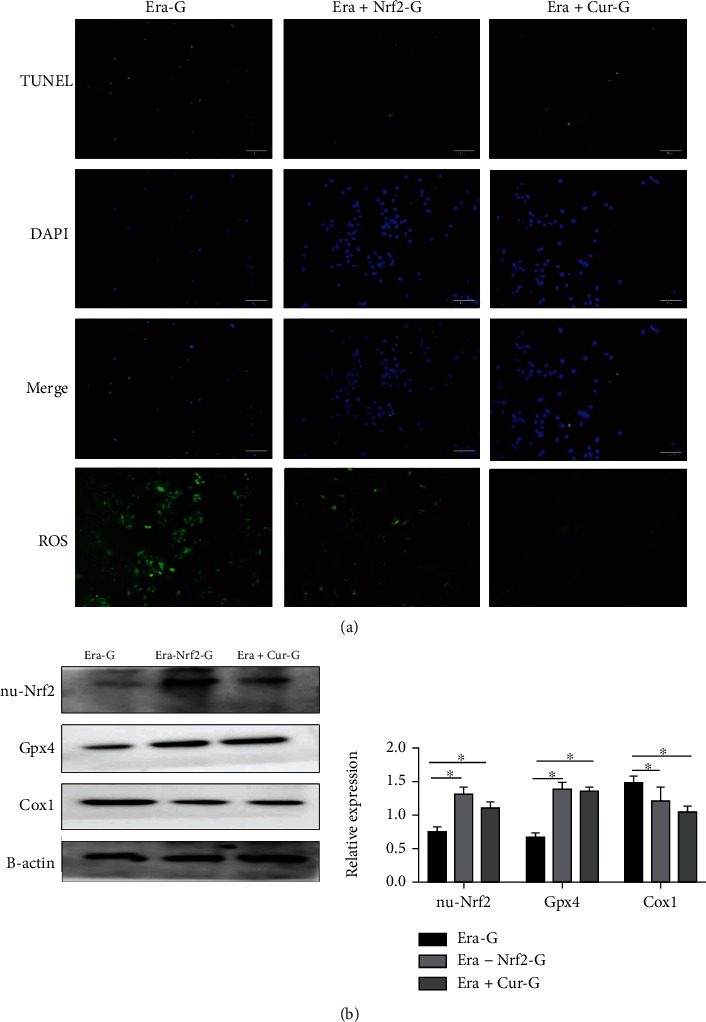
Curcumin prevents cardiac damage caused by erastin. Era-G: erastin-treated cardiomyocytes; Era+Nrf2-G: Nrf2 overexpressing+erastin-treated cardiomyocytes; Era+Cur-G: curcumin+erastin-treated cardiomyocytes. (a) Terminal deoxynucleotidyl transferase dUTP nick-end labelling (TUNEL) and reactive oxygen species (ROS) assays revealed that ferroptosis inducers caused massive myocardial cell death. The apoptosis rate and intracellular ROS were significantly reduced vs. the Era-G cells (×200). (b) Western blot detected the expression levels of nuclear Nrf2 and ferroptosis-related factors (^∗^*P* < 0.05).

The ferroptosis inducer erastin was used to treat H9C2 cardiomyocytes, and the dose of erastin was set at 1 mmol/L based on relevant literature [12]. After 24 h of cocultivation, erastin was added to Nrf2-overexpressing cardiomyocytes and curcumin-treated cardiomyocytes. The results showed that when cardiomyocytes were treated with erastin, ROS levels significantly increased while cell viability significantly decreased (Figure 4(a)). Cardiomyocyte curcumin treatment and Nrf2 overexpression increased the expression of Gpx4 in the cytoplasm and Nrf2 in the nucleus and reduced ROS accumulation and ferroptosis-induced cell damage (Figure 4(b)).

## 4. Discussion

Elevated blood sugar causes cardiomyocyte insulin resistance, cellular lipid peroxidation, activation of inflammatory factors, cellular microenvironments of oxidative stress and inflammatory factors, RASS, and sympathetic nerve stimulation activation. All these result in myocardial fiber changes and decreased myocardial function [13–15]. Myocardial slices and collagen staining showed that diabetes caused cardiomyocyte hypertrophy, disordered cell arrangements, and increased myocardial fibrosis. Furthermore, diabetes decreased myocardium Gpx4 and nuclear Nrf2 expression, increased the expression of downstream-related ferroptosis factors, and increased superoxide accumulation, demonstrating that ferroptosis is closely related to diabetic cardiomyopathy.

Ferroptosis is a process in which cells are stimulated to cause excessive intracellular accumulation of iron ions and lipid peroxides resulting in oxidative stress and cell death [16]. Park et al. discovered that reducing Gpx4 expression in myocardial infarction mouse models resulted in a worsening of myocardial cell ferroptosis and impaired myocardial cell activity [17]. This experiment demonstrated that at the animal and cellular levels, glucose-induced continuous cardiomyocyte peroxide accumulation triggers ferroptosis and causes cell damage. Curcumin was discovered to increase the expression of liver insulin-degrading enzymes and maintain the integrity of islets as an oxygen free radical scavenger [18, 19]. According to Ali's research, curcumin can counteract D-galactose-induced oxidative stress in rat brain and heart by lowering Bax and CASP-3 expression. This is a common misunderstanding [20]. This study showed that curcumin promotes the transfer of Nrf2 into the nucleus, increases the expression of antioxidant factor HO-1, reduces cardiomyocyte ROS accumulation, and reduces the degree of ferroptosis caused by high glucose levels.

By regulating the expression of glutathione peroxidase, glutathione s-transferase, and glutathione reductase, Nrf2 maintains reduced levels of intracellular glutathione and cellular redox homeostasis [21, 22]. When cells are disrupted by ROS, Nrf2 translocates to the nucleus and binds to the antioxidant response element/electrophilic response element (ARE/EpRE) of target genes via heterodimerization alongside somite Maf protein which induces the expression of downstream protective genes [23, 24]. Furthermore, Li discovered that curcumin reduces mercury-induced stem cell damage by promoting Nrf2 nucleus translocation [25]. This experiment found that curcumin promotes the transfer of Nrf2 into the nucleus, prompts cells to express a large number of antioxidant factors including HO-1, reduces the excessive downregulation of Gpx4, and inhibits the progression of cell ferroptosis. Curcumin can also reduce erastin-induced ferroptosis, accumulation of oxidative products, and degree of cell damage. Although further studies are required, the results of this study may provide a new therapeutic direction for the treatment of diabetic cardiomyopathy.

## 5. Conclusions

Diabetic cardiomyopathy can cause heart vessel blockages, myocardial contractile dysfunction, and a significant reduction in quality of life. The pathogenesis of diabetic cardiomyopathy is not entirely known as well as what the most effective prevention and treatment methods are.

Curcumin has been shown to have antioxidant properties and to maintain cell activity. However, its effect on diabetic cardiomyopathy has not yet been established. This study found that feeding diabetic rabbits curcumin significantly improved myocardial structure, including myocardial fibrosis and cell arrangement disorder. The therapeutic effect of curcumin significantly improved as dosing frequency increased. Curcumin was also found to increase Nrf2 transfer into the nucleus, increase the expression of Gpx4 and HO-1, reduce glucose-induced myocardial cell damage, and reverse myocardial cell damage caused by erastin. This study confirmed the role of ferroptosis in the pathogenesis of diabetic cardiomyopathy and provided a new theoretical direction for diabetic cardiomyopathy prevention and treatment.

## Data Availability

The data covered in this article are included in the article.

## References

[1] Crasto W., Patel V., Davies M. J., Khunti K. (2021). Prevention of microvascular complications of diabetes. *Endocrinology and Metabolism Clinics of North America*.

[2] Murtaza G., Virk H. U. H., Khalid M. (2019). Diabetic cardiomyopathy - a comprehensive updated review. *Progress in Cardiovascular Diseases*.

[3] Sun Y., Chen P., Zhai B. (2020). The emerging role of ferroptosis in inflammation. *Biomedicine & Pharmacotherapy*.

[4] Bruni A., Pepper A. R., Pawlick R. L. (2018). Ferroptosis-inducing agents compromise in vitro human islet viability and function. *Cell Death & Disease*.

[5] Tong M., Saito T., Zhai P. (2019). Mitophagy is essential for maintaining cardiac function during high fat diet-induced diabetic cardiomyopathy. *Circulation Research*.

[6] Nakamura M., Sadoshima J. (2020). Cardiomyopathy in obesity, insulin resistance and diabetes. *The Journal of Physiology*.

[7] White C. M., Pasupuleti V., Roman Y. M., Li Y., Hernandez A. V. (2019). Oral turmeric/curcumin effects on inflammatory markers in chronic inflammatory diseases: a systematic review and meta-analysis of randomized controlled trials. *Pharmacological Research*.

[8] Rainey N. E., Moustapha A., Petit P. X. (2020). Curcumin, a multifaceted hormetic agent, mediates an intricate crosstalk between mitochondrial turnover, autophagy, and apoptosis. *Oxidative Medicine and Cellular Longevity*.

[9] Wang Y., Liu F., Liu M. (2022). Curcumin mitigates aflatoxin B1-induced liver injury via regulating the NLRP3 inflammasome and Nrf2 signaling pathway. *Food and Chemical Toxicology*.

[10] Li J., Lu K., Sun F. (2021). Panaxydol attenuates ferroptosis against LPS-induced acute lung injury in mice by Keap1-Nrf2/HO-1 pathway. *Journal of Translational Medicine*.

[11] Ren J., Su D., Li L. (2020). Anti-inflammatory effects of Aureusidin in LPS-stimulated RAW264.7 macrophages via suppressing NF-*κ*B and activating ROS- and MAPKs-dependent Nrf2/HO-1 signaling pathways. *Toxicology and Applied Pharmacology*.

[12] Bai Y. T., Xiao F. J., Wang H., Ge R. L., Wang L. S. (2021). Hypoxia protects H9c2 cells against ferroptosis through SENP1-mediated protein DeSUMOylation. *International Journal of Medical Sciences*.

[13] Wang X., Pan J., Liu D. (2019). Nicorandil alleviates apoptosis in diabetic cardiomyopathy through PI3K/Akt pathway. *Journal of Cellular and Molecular Medicine*.

[14] Yan D., Cai Y., Luo J. (2020). FOXO1 contributes to diabetic cardiomyopathy via inducing imbalanced oxidative metabolism in type 1 diabetes. *Journal of Cellular and Molecular Medicine*.

[15] Dhalla N. S., Shah A. K., Tappia P. S. (2020). Role of oxidative stress in metabolic and subcellular abnormalities in diabetic cardiomyopathy. *International Journal of Molecular Sciences*.

[16] Lee S. J., Chandrasekran P., Mazucanti C. H., O’Connell J. F., Egan J. M., Kim Y. (2022). Dietary curcumin restores insulin homeostasis in diet-induced obese aged mice. *Aging (Albany NY)*.

[17] Park T. J., Park J. H., Lee G. S. (2019). Quantitative proteomic analyses reveal that GPX4 downregulation during myocardial infarction contributes to ferroptosis in cardiomyocytes. *Cell Death & Disease*.

[18] Abu-Taweel G. M., Attia M. F., Hussein J. (2020). Curcumin nanoparticles have potential antioxidant effect and restore tetrahydrobiopterin levels in experimental diabetes. *Biomedicine & Pharmacotherapy*.

[19] Gutierres V. O., Assis R. P., Arcaro C. A. (2019). Curcumin improves the effect of a reduced insulin dose on glycemic control and oxidative stress in streptozotocin-diabetic rats. *Phytotherapy Research*.

[20] El-Far A. H., Elewa Y. H., Abdelfattah E. Z. (2021). Thymoquinone and curcumin defeat aging-associated oxidative alterations induced by D-galactose in rats’ brain and heart. *International Journal of Molecular Sciences*.

[21] Lv H., Yang H., Wang Z. (2019). Nrf2 signaling and autophagy are complementary in protecting lipopolysaccharide/d-galactosamine-induced acute liver injury by licochalcone A. *Cell Death & Disease*.

[22] Uruno A., Matsumaru D., Ryoke R. (2020). Nrf2 Suppresses Oxidative Stress and Inflammation inAppKnock-In Alzheimer’s Disease Model Mice. *Molecular and Cellular Biology*.

[23] Ye W., Ma J., Wang F. (2020). LncRNA MALAT1 regulates miR-144-3p to facilitate epithelial-mesenchymal transition of lens epithelial cells via the ROS/NRF2/Notch1/snail pathway. *Oxidative Medicine and Cellular Longevity*.

[24] Yu C., Xiao J. H. (2021). The Keap1-Nrf2 system: a mediator between oxidative stress and aging. *Oxidative Medicine and Cellular Longevity*.

[25] Li S., Wang X., Xiao Y. (2021). Curcumin ameliorates mercuric chloride-induced liver injury via modulating cytochrome P450 signaling and Nrf2/HO-1 pathway. *Ecotoxicology and Environmental Safety*.

